# Insights into Preparation Methods and Functions of Carbon-Based Solid Acids

**DOI:** 10.3390/molecules29010247

**Published:** 2024-01-03

**Authors:** Dong Shu, Jian Zhang, Roger Ruan, Hanwu Lei, Yunpu Wang, Qian Moriko, Rongge Zou, Erguang Huo, Dengle Duan, Lu Gan, Dan Zhou, Yunfeng Zhao, Leilei Dai

**Affiliations:** 1 Key Laboratory of Agricultural Product Processing and Quality Control of Specialty (Co-Construction by Ministry and Province), School of Food Science and Technology, Shihezi University, Shihezi 832003, China; 20212111016@stu.shzu.edu.cn (D.S.); zhangjian0411@163.com (J.Z.); 20201011067@stu.shzu.edu.cn (L.G.); m17590391085@163.com (D.Z.); 2 Key Laboratory for Food Nutrition and Safety Control of Xinjiang Production and Construction Corps, School of Food Science and Technology, Shihezi University, Shihezi 832003, China; 3 Center for Biorefining and Department of Bioproducts and Biosystems Engineering, University of Minnesota, 1390 Eckles Ave., St. Paul, MN 55112, USA; ruanx001@umn.edu; 4 Department of Biological Systems Engineering, Washington State University, Richland, WA 99354, USA; hlei@wsu.edu (H.L.); moriko.qian@wsu.edu (Q.M.); rongge.zou@wsu.edu (R.Z.); 5 State Key Laboratory of Food Science and Technology, Nanchang University, Nanchang 330047, China; wangyunpu@ncu.edu.cn; 6 Jiangsu Key Laboratory of Micro and Nano Heat Fluid Flow Technology and Energy Application, School of Physical Science and Technology, Suzhou University of Science and Technology, Suzhou 215009, China; huoerguang@126.com; 7 Guangdong Provincial Key Laboratory of Lingnan Specialty Food Science and Technology, Zhongkai University of Agriculture and Engineering, Guangzhou 510225, China; duandenglele@zhku.edu.cn

**Keywords:** carbon-based solid acid, preparation method, carbonization method, acidification type, function

## Abstract

With the growing emphasis on green chemistry and the ecological environment, researchers are increasingly paying attention to greening materials through the use of carbon-based solid acids. The diverse characteristics of carbon-based solid acids can be produced through different preparation conditions and modification methods. This paper presents a comprehensive summary of the current research progress on carbon-based solid acids, encompassing common carbonization methods, such as one-step, two-step, hydrothermal, and template methods. The composition of carbon source material may be the main factor affecting its carbonization method and carbonization temperature. Additionally, acidification types including sulfonating agent, phosphoric acid, heteropoly acid, and nitric acid are explored. Furthermore, the functions of carbon-based solid acids in esterification, hydrolysis, condensation, and alkylation are thoroughly analyzed. This study concludes by addressing the existing drawbacks and outlining potential future development prospects for carbon-based solid acids in the context of their important role in sustainable chemistry and environmental preservation.

## 1. Introduction

Amidst the escalating environmental concerns, the chemical industry is increasingly demanding more environmentally friendly processes. To this end, Professor Anastas of the *United States Environmental Protection Agency* proposed the concept of *green chemistry* [1]. *Green chemistry* is the design of chemical products and processes that reduce or eliminate the use and generation of hazardous substances. In the chemical industry, green chemistry can make the best of raw materials and reduce the production of harmful substances and environmental pollution. As one of the methods conforming to the definition of *green chemistry*, catalysis plays an important role in reducing environmental pollution [2,3,4]. Catalysis makes the reaction more efficient, thereby contributing to the elimination of harmful substances.

In the field of catalysis, acid catalysis is one of the most widely used catalytic methods in the chemical industry [5]. Traditional acid catalysis primarily relies on liquid acids like sulfuric acid [6], nitric acid [7], or hydrochloric acid [8] to catalyze various reactions. However, the use of conventional liquid acids poses potential issues such as the corrosion of reaction equipment, challenges in product separation, emission of environmental pollutants, and high costs associated with waste disposal [9]. These drawbacks contradict the principles of green chemistry. Compared to liquid acids, solid acids have high temperature resistance, are easy to separate, are less polluting, and are reusable [10,11]. The trend of replacing liquid acids with solid acids is gaining momentum to achieve environmentally friendly catalytic processes.

Solid acids are prepared by loading acid groups onto suitable carrier materials. Therefore, the performance of solid acids is closely related to the strength and quantity of acid sites and the morphology of carrier (specific surface area and porosity) [12,13]. Carbon-based solid acids represent are novel and highly efficient catalysts, primarily developed by incorporating acidic functional groups into carbon materials. Compared with other catalysts, this type of catalyst offers the following advantages [14,15]:

Rich acidic groups, such as sulfonic acid groups, hydroxyl groups, and carboxyl groups;
Good biocompatibility via reduced toxic residuals compared with metal-based catalysts;
Rich in raw materials and low in cost (carbon precursors are often animal wastes or plant wastes);
Easy recycling and minimal corrosion of equipment.

The current research has focused on the preparation methods and functions of carbon-based solid acids. Carbon-based solid acids are often used in hydrolysis, alkylation, condensation, esterification, and transesterification [16,17,18,19,20]. For the preparation of carbon-based solid acid catalysts, the carbonization method and the type of reagents in the acidification process are the main factors affecting the physical and chemical properties of the catalyst. However, at present, most of the reviews on solid acids have analyzed the differences between different solid acids from the perspective of application, such as the production of biodiesel and the preparation of 5-hydroxymethylfurfural [21,22,23,24]. Few reviews have discussed carbonization methods and acidification types separately. Therefore, this paper aims to summarize the state of the art in carbonization methods and acidification types of carbon-based solid acid catalysts so as to provide guidance for further research on carbon-based solid acid catalysts.

## 2. Research on Different Carbonization Methods for Carbon-Based Solid Acids

The physical and chemical properties of carbon-based solid acids vary with different carbonization methods. Since various carbonization methods result in alterations to the morphology of carbon-based solid acid catalysts, carbon pore density, pore size, and the tightness of the catalytic acidic groups on the carbon matrix, they consequently impact the properties and acidity of the carbon-based solid acid catalyst. The carbonization process is generally divided into two stages: firstly, at low temperature, biomass is dehydrated, decarboxylated, and dehydrogenated to remove volatile components; secondly, with the increase of temperature, a large number of hydrocarbons are converted into solid substances and volatile gases through cracking, polycondensation, and other reactions. Volatile substances are removed with inert gas flow. With the removal of volatile substances (H_2_O, CO_2_, CH_4_, CO, etc.), the carbon content gradually increases, and finally carbon-based materials are generated. At present, the mainstream carbonization methods of carbon-based solid acid include one-step carbonization, two-step carbonization, and precise targeting method (hydrothermal and template methods). 

### 2.1. One-Step Carbonization

One-step carbonization involves placing the carbon source material in sulfonating agent at a certain temperature [25,26,27]. The method has the advantages of simplifying the process steps, reducing the preparation time of the carbon-based solid acid, and reducing the material cost [28,29]. The method was first proposed by Hara et al. [30]. Under a nitrogen atmosphere, naphthalene and concentrated sulfuric acid were mixed and heated with continuous stirring for 15 h, yielding an acidic carbon-based material with an acid content as high as 4.9 mmol·g^−1^. NMR analysis revealed that sulfonic acid groups could be successfully introduced onto aromatic carbon atoms. The catalytic performance of this type of acidic carbon-based material in catalyzing ethyl acetate synthesis is significantly better than that of traditional solid acids such as perfluorosulfonic acid resins. However, when the liquid phase reaction temperature of this kind of carbon-based solid acid is higher than 200 °C or the reactant has a higher fatty acid content, the acid centers are prone to detachment, resulting in rapid catalyst deactivation. The main reason for catalyst deactivation is that when the reaction temperature is too high, sulfur leaching leads to the deactivation of carbon-based solid acids. When the reactant has a higher fatty acid content, the formation of sulfonate leads to the deactivation of carbon-based solid acids.

**Figure 1:**
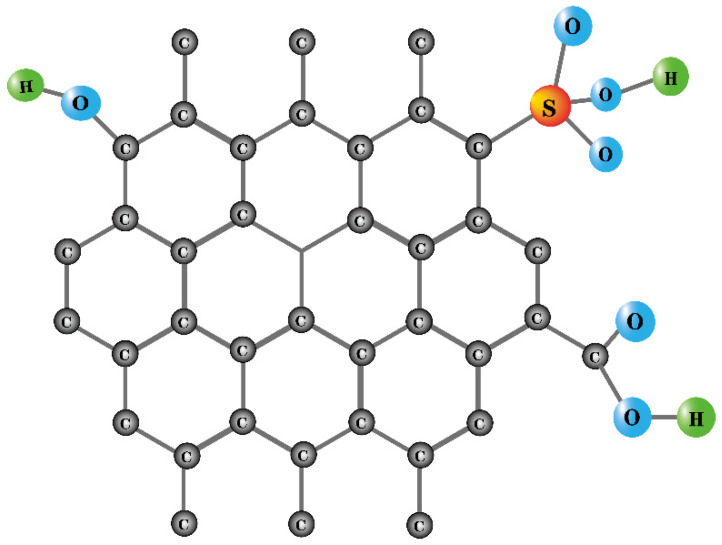
Schematic diagram of Surface distribution of acidic groups in carbon-based solid [31].

Kang et al. [31] used red liquid solid, which is a by-product of the papermaking process (a phenolic polymer whose main organic component is lignosulfonate) as the carbon precursor and concentrated sulfuric acid as the sulfonating reagent to prepare a solid acid catalyst. Compared with traditional solid acid catalysts (strong acid 732 cation exchange resin, hydrogen zeolite HZSM-5, and zirconium sulfate), the carbon-based solid acid catalyst prepared using this method showed higher and more stable catalytic activity in oleic acid esterification. The possible reason is that traditional solid acid catalysts usually contain a single acidic functional group, while carbon-based solid acid catalysts have three different acidic functional groups (–OH, –COOH, and –SO_3_H), as shown in Figure 1 [31]. At the same time, carbon-based solid acids have a large content of –OH groups (2.18 mmol·g^−1^), which can attach two polar reactants (methanol and oleic acid) to the catalyst surface and then accelerate the esterification reaction. These results also indicate that the carbon-based sulfonated catalysts synthesized through the direct sulfonation and carbonization of macromolecule polymers may exhibit greater stability in comparison to those derived from low-molecular-mass compounds, aligning with the observations made by Hara et al. [30].

Mateo et al. [32] used a carbon-based solid acid to catalyze the delignification of organic solvents for the first time. Carbon-based solid acid catalysts facilitate the release of lignin components from lignocellulosic biomass by promoting the cleavage of lignin–carbohydrate bonds, with the additional presence of ethanol aiding in lignin dissolution. In addition, Liang et al. [33] used furfural and isethionic acid as raw materials to synthesize a new type of carbon-based solid acid containing sulfonic acid groups via direct carbonization and sulfonation under mild hydrothermal conditions. The solid acid product, which was used to catalyze the esterification of acetic acid and butanol, exhibited comparable catalytic activity to sulfuric acid. Additionally, Zhang et al. [34] synthesized magnetic carbon-based solid acid from chitosan, magnetic core, and hydroxyethyl sulfonic acid via direct carbonization and sulfonation under the same conditions. The material exhibited a high specific surface area and a core–shell structure, which provided accessible acid sites for the carbon shell. In terms of catalytic performance, the new magnetic carbon-based solid acid shows high activity for the hydrophobic alkylation reaction of 1-dodecene and benzene, which is not easily activated using traditional carbon-based solid acids. In addition, it can make the best of the magnetic properties of solid acids, contributing to the separation of solid acid and product. 

Currently, the predominant approach for simplifying the preparation of carbon-based solid acids is the one-step method, involving carbonization and sulfonation. While offering benefits such as speed and energy efficiency, the one-step carbonization and sulfonation method may not be the optimal approach for preparing solid acids using carbon-based raw materials from diverse sources. This is because carbon-based solid acid catalysts prepared via the direct sulfonation and carbonization of macromolecules tend to exhibit greater stability compared to those prepared from low-molecular-weight compounds. Simultaneously, this method presents various challenges related to safety and large-scale production. These issues arise from the carbonization of the sulfoaromatic ring within concentrated sulfuric acid at temperatures exceeding 200 °C [35]. Therefore, it is crucial to choose different solid acid preparation methods based on the specific requirements and the inherent characteristics of the raw materials.

Cao et al. [36] used a solid acid catalyst prepared from *Sargassum algae* for the esterification of oleic acid and methanol to produce biodiesel. The results showed that the carbonization temperature affected the final size of the amorphous carbon sheet and the degree of the graphite-like phase, while the carbonization time determined whether the amorphous aromatic ring carbon sheet formed completely. In addition, the specific surface area of carbon-based materials increased by nearly four times after phosphoric acid activation. In an attempt to achieve a circular bioeconomy in microalgae biodiesel production, Roy et al. [37] harnessed catalysts derived from processed microalgae to facilitate the catalysis of microalgal lipids into biodiesel. In the preparation process, it was found that the biochar was hydrophobic; the hydrophobic nature of the biochar could be attributed to the alkyl functional groups on its surface. Hydrophobic carbon acts as potential support for sulfonated carbon catalysts as it prevents the -OH and -SO_3_H groups from being hydrated by the water generated during the esterification process. Hydrophobic supports also repel the glycerol produced during the transesterification reactions from the active sites of the catalyst, making them solely available for the reactants. Thus, hydrophobic carbon supports maintain catalyst stability and enhance catalyst reusability. Wang et al. [38] used starch as carbon precursor, first mixed starch with KOH for precarbonization, then performed carbonization at high temperature, and finally conducted sulfonation with sulfuric acid. Through this method, the solid acid effectively adsorbed chitosan molecules via –OH and –COOH groups, thereby facilitating the action of –SO_3_H in breaking glycosidic bonds. Sangar et al. [39] used glycerol as a carbon precursor and sulfuric acid as a sulfonating agent. The solid acid prepared with this method showed good catalytic performance in catalyzing the conversion of palm fatty acid and methanol into biodiesel.

Although carbonization followed by sulfonation is the most commonly used preparation method for carbon-based solid acid catalysts [40,41], it has some disadvantages. For example, when the temperature rises unevenly in the carbonization stage, it causes the uneven distribution of acid sites and groups; when the temperature is too low, the carbocyclic ring is broken due to side reactions, such as oxidation in the subsequent sulfonation process, causing structural instability and easy fusion with polar solvents; when the carbonization temperature is too high, the structural rigidity of carbon-based materials increases, and the amorphous carbon content decreases, which lead to the decrease in the number of sulfonation sites of sulfonating agents, thus reducing the acidity of carbon-based solid acids and making them more easily inactivated [42,43,44].

For the aforementioned reasons, some researchers have proposed a method of sulfonation followed by carbonization to prepare carbon-based solid acids. The method of sulfonation followed by carbonization usually uses fused-ring aromatic compounds as carbon precursors for sulfonation and then incomplete carbonization. Sangar et al. [45] used hollow palm fruit string as a carbon precursor, performed sulfonation in concentrated sulfuric acid, then conducted carbonization at high temperature to obtain the required carbon-based solid acid. The carbon-based solid acid catalyst showed good performance in the reaction of preparing biodiesel; it still had considerable catalytic activity after six reuses. Hence, during the procedure of stepwise carbonization and sulfonation for solid acid preparation, careful consideration of the raw material’s structure and intended final application remains essential. The selection of an appropriate preparation method should be based on varying application conditions [46,47]. For example, when a solid acid is to be used in a relatively mild environment such as in hydrolysis and esterification, the preparation method of carbonization first and then sulfonation can be used. If the solid acid is to be applied in a relatively extreme reaction environment such as the conversion of pyrolysis gas, the preparation method of sulfonation first and then carbonization can be considered. 

### 2.2. Precise Targeting Methods

The carbon-based solids acid prepared via the step method have high stability. However, higher carbonization temperature leads to the reduction of organic matter and the overall acidity of solid acid. Traditional carbonization methods mainly aim at relatively complex biomass-based materials. For carbon source materials from a single source (glucose, sucrose, starch, etc.), a more refined directional carbonization method can be used. Utilizing directional carbonization can confer favorable carrier properties upon carbon-based materials, including uniform pore diameter and a high specific surface area. Consequently, acid groups can be more effectively incorporated onto carbon-based materials, enabling the production of carbon-based solid acids with heightened acidity. These methods are mainly categorized as hydrothermal carbonization [48,49] or template carbonization [50,51].

#### 2.2.1. Preparation of Carbon-Based Solid Acid by Hydrothermal Carbonization

Some researchers have proposed a hydrothermal carbonization method, leveraging the unique characteristic of solutions in the critical state to diminish the energy demand for the reaction. This method mainly involves heating an aqueous solution within a specific closed reactor (hydrothermal kettle), under a controlled-pressure environment. This process propels the solution system into a critical state. In this state, carbon precursor materials undergo dehydration and condensation, leading to the formation of intermediates such as furans, aldehydes, or phenols. With the increase in temperature, the color of the solution becomes darker, the viscosity increases, and the molecules polymerize again to form crystal nuclei. With the growth of crystal nuclei, it gradually carbonizes [52,53,54]. Notably, this process is influenced by the gradual formation and subsequent carbonization of these crystal nuclei. Given the limited solubility of lignin, plant matter, and other primitive biomass in water, as opposed to the water solubility of its sugar derivatives, most research on biomass hydrothermal carbonization has used sugar as carbon source. Compared with the traditional carbon-based solid acid preparation method, the hydrothermal carbonization method boasts several advantages, including relatively lower reaction temperature, shorter reaction time, higher carbonization yield, and reduced production of waste gases and liquids.

Fu et al. [55] prepared a carbon-based solid acid catalyst through hydrothermal carbonization, with β-cyclodextrin and concentrated sulfuric acid as the carbon precursor and sulfonating agent, respectively. During the hydrothermal carbonization stage, researchers analyzed the carbonization products of β-cyclodextrin with water, dilute sulfuric acid, and the sulfonated carbonation products of concentrated sulfuric acid through scanning electron microscopy, and they concluded that the addition of sulfuric acid can accelerate β-cyclodextrin carbonization. The carbonization of cyclodextrin increases the particle size of its spherical structure and provides more sites for the anchoring of sulfonic acid groups. The solid acid obtained with this approach exhibits a higher density, and its catalytic activity is much higher than that of the commonly used amberlyst-15 solid acid catalyst in the catalytic reaction of oleic acid esterification. It still had considerable catalytic activity after being reused six times. Wang et al. [56] designed a strategy of combining oxidation, amidation, and sulfonation to prepare carbon-based solid acid catalysts with high acid density. Firstly, glucose was hydrothermally carbonized into carbon-based materials, and then the carbon-based materials were oxidized to increase the carboxyl content. P-phenylenediamine was introduced to the surface of carbon-based materials via an amidation reaction with carboxyl group on the surface of carbon-based materials, which provides a reactant for the chemical bonding of sulfonic acid groups on the surface of carbon-based materials. P-phenylenediamine on the surface of carbon-based materials reacts with chlorosulfonic acid through sulfonation reaction. Finally, a carbon-based solid acid catalyst with an acid density as high as 11.31 mmol·g^−1^ was synthesized. The carbon-based solid acid was applied to catalytic conversion of frying waste oil into biodiesel, and the conversion rate reached 92.2%. 

**Figure 2:**
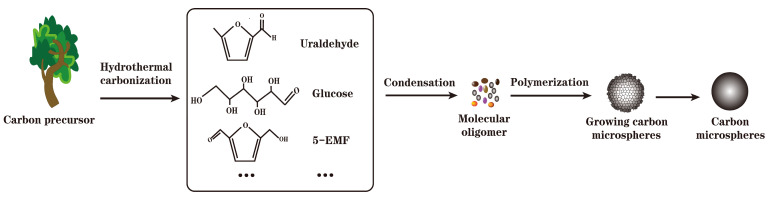
Formation mechanism of hydrothermal carbonized carbon microspheres.

The reason why the microstructure of carbon-based materials is spherical after hydrothermal carbonization is shown in Figure 2. Under hydrothermal conditions, the dehydration and condensation between glucose molecules lead to the polymerization of long-chain oligosaccharides and aromatic compounds. When the concentration of oligosaccharide molecules in the solution reaches the critical saturation state, the dehydration reaction between oligosaccharide molecules and aromatic compounds occurs again, leading to cross-linking and polymerization and the forming of a spherical structure with hydrophobic groups as the core and hydrophilic groups as the outer surface. There are irregular aromatic ring structures in the spherical structure [57]. Furthermore, the carbon framework generated through this approach exhibits a comparatively porous structure and a lower level of graphitization. This characteristic yields an abundance of acidic sites conducive to sulfonation, while its surface features a substantial presence of oxygen-containing groups, facilitating chemical modifications. The carbonization temperature and carbonization time of carbon-based solid acids prepared via hydrothermal carbonization are lower than those of traditional carbonization [58,59]. Based on its advantages of low energy and convenience, more and more studies are being conducted on the preparation of carbon-based solid acids through this method [60].

#### 2.2.2. Template Carbonization

The biggest difference between template carbonization and other carbonization methods lies in its capacity to induce a well-defined pore structure in carbon materials through the guiding effect of the template mechanism. This method is often used for the creation of mesoporous carbon materials. Based on the template used, these methods are categorized as hard template [61] or soft template [62] methods.

The hard template method is also called the nano-casting method. In this method, the precursor of the target material is cast into the nano channel of a rigid inorganic solid template, and then the precursor is transformed into the desired product via heating or other treatment. Finally, the template material is removed to obtain a material with a regular channel structure. Removal methods generally include extraction, roasting, microwave digestion, or irradiation. In the preparation of carbon-based solid acids using the hard template method, soluble substances such as sucrose and phenolic resin are generally used as carbon sources, and cheap materials such as silica gel, silica, or alumina films are used as template materials. Yang et al. [63] used zeolite as a template, cast carbon precursors into the template via infiltration and chemical vapor deposition (CVD), and successfully synthesized a three-dimensional ordered microporous carbon material with uniform pores. Wang et al. [64] used the porous silicon material TUD-1 as a hard template, and they used flowing CH_4_ as a carbon precursor, carbonized at a high temperature of 900 °C, and finally removed the template with an HF solution. The carbon material prepared with this method has an irregular sponge-like structure similar to the TUD-1 template. Lin et al. [64] used sucrose as the carbon precursor and cage-like mesoporous silica (KIT-5-150) as the hard template to synthesize a carbon material with a specific surface area of 1368 m^2^·g^−1^, sulfonating with benzene sulfonic acid to obtain a carbon-based solid acid catalyst. Yang et al. [65] used sucrose as the carbon precursor and all-silicon SBA-15 synthesized with triblock polymer (P123) and tetraethyl orthosilicate (TEOS) as the hard template. After the two were mixed and carbonized, hydrogen fluoric acid removed the template, and the obtained carbon materials were, respectively, placed in concentrated sulfuric acid and p-aminobenzenesulfonic acid for sulfonation to obtain two types of carbon-based solid acids. The specific surface areas of the two carbon-based solid acids were 924 m^2^·g^−1^ and 1001 m^2^·g^−1^; the surface acid density was around 4.0 mmol·g^−1^. In the epoxidation reaction of catalyzing the conversion of waste frying oil into epoxides, the conversion rates of the two could reach 77.2% and 68.5%, respectively; the maximum conversions were 90.3% and 89.0% in the reaction of using waste frying oil to produce biodiesel.

**Figure 3:**
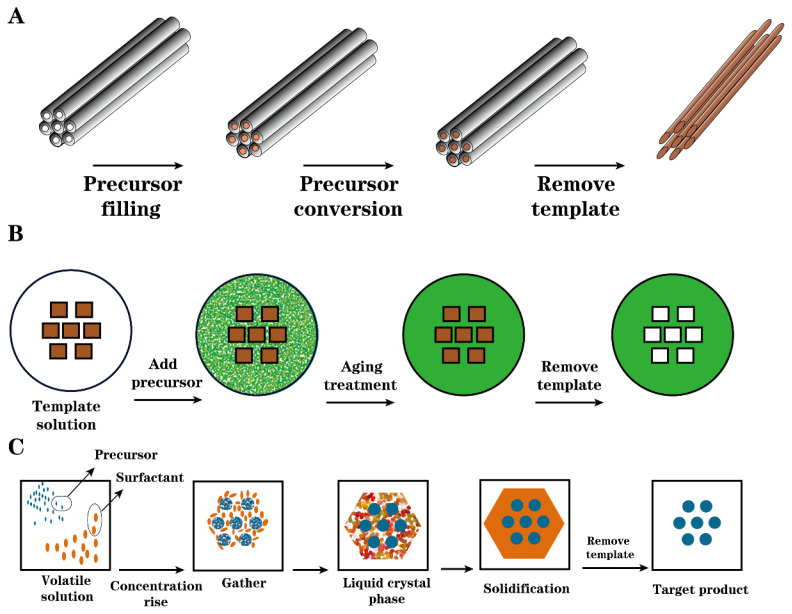
Mesoporous structure synthesis pathway (**A**) hard template, (**B**) liquid crystal template (LCT), and (**C**) collaborative self assembly (CSA) [10].

Although the mesoporous carbon-based solid acids prepared via the hard template process have a regular pore size that contributes to the uniform distribution of acid sites, there are many disadvantages in the preparation process, such as a longer synthesis time and the loss of template content. Compared with the hard template, although the soft template cannot control the structural framework of the carbon material, the carbon materials prepared via the soft template have a lower pore volume and a more stable structure. At the same time, it has the advantages of strong repeatability and simple operation [66,67,68]. The soft template method mainly uses the noncovalent bonds (such as van der Waals force, hydrogen bond, electrostatic attraction, and hydrophobic bond) between organic or inorganic molecules and surfactant molecules to spontaneously form thermodynamically stable and ordered supramolecular structures. To prepare the desired material, the template agent can be a surfactant, silicone, or block copolymer. According to the different media used in the synthesis, the soft template method is categorized liquid crystal template (LCT) and cooperative self-assembly (CSA).With the liquid crystal template method, = the template agent is first dissolved in water, the pH is adjusted to an appropriate value, the precursor is added, and a precipitate is obtained after a sol–gel reaction, which is then placed it at room temperature or higher temperature (hydrothermal). After aging treatment, the template is finally removed to obtain the target product; cooperative self-assembly involves the slow volatilization of nonaqueous solvents to increase the concentration of the solution phase composed of precursors and surfactant molecules, making it into a liquid crystal phase, and then drying and curing. Finally, the templating agent is removed. The three approaches of the template method are shown in Figure 3 [10].

A researcher [69] used triblock copolymer F127 as a template agent and resorcinol-formaldehyde as a carbon precursor to prepare carbon materials via the liquid crystal template method. According to instrumental analysis, its specific surface area was about 697 m^2^·g^−1^. In addition, Kevin et al. [70] used glucose as the carbon precursor and sodium bicarbonate as the template, mixed them to form a uniform solution, removed the water on a rotary evaporator, and finally formed a uniformly mixed solid. Then, the mixture was transferred to a quartz tube furnace, carbonization was carried out under the protection of nitrogen, and then sodium bicarbonate was removed using hydrochloric acid to obtain a porous carbon intermediate. Finally, the carbon intermediate was sulfonated using concentrated sulfuric acid to prepare a carbon-based solid acid. Wang et al. [71] used kraft lignin as the carbon precursor and triblock copolymer F127 as the template, mixed the two, placed them in a tube furnace, carbonized them under the protection of nitrogen, and then sulfonated them in sulfuric acid to obtain a carbon-based solid acid. The solid acid had a specific surface area of 262 m^2^·g^−1^; it demonstrated quite good catalytic activity in the reaction of catalyzing fructose to 5-hydroxymethylfurfural (5-HMF).

For both the hydrothermal method and the template method, carbon precursors need to be prepared in advance in the process of preparing carbon-based solid acids, and the preparation conditions of carbon precursors directly determines the acidity of a carbon-based solid acid. The comprehensive research results show that precise targeting methods (hydrothermal carbonization method and template method) can increase the effective area of amorphous carbon and make carbon-based solid acid have strong acidity and catalytic activity. However, the process of preparing carbon-based solid acids with this method is quite expensive, and it is often only for a certain type of reaction. 

In Table 1, we summarize the raw materials, carbonization methods, and functions for the preparation of carbon-based solid acids. As shown in this table, that different raw materials correspond to different carbonization temperatures. The reason may be related to the composition of the raw materials. Although there are various types of biomass raw materials, they are all composed of cellulose, hemicellulose, and lignin. Researchers obtained a conclusion through thermogravimetric mass spectrometry: hemicellulose and cellulose, with a large number of functional groups such as hydroxyl groups, have lower pyrolysis temperatures, while lignin, with a large number of stable aromatic groups, needs higher pyrolysis temperatures [72]. Therefore, for biomass with a high lignin content, a higher carbonization temperature is needed to carbonize it. In addition, according to this table, we can find that in most cases, carbon-based solid acid catalysts prepared via direct carbonization (one-step carbonization, two-step carbonization, or hydrothermal carbonization) have smaller specific surface area and pore size. However, the template method can overcome this limitation.

## 3. Different Acidification Methods of Carbon-Based Solid Acids

The common acidification methods mainly include sulfonating agent sulfonation, phosphoric acid acidification, heteropoly acid acidification, and nitric acid acidification. Table 2 summarizes the acidification methods, raw materials, and applications of carbon-based solid acids. It can be seen from Table 2 that the influences of acidification temperature and acid concentration on the acid catalytic performance of carbon-based solid acids are greater than that of acidification time, because acidification temperature and acid concentration may change the combination mode of acid groups and amorphous carbon.

### 3.1. Preparation of Carbon-Based Solid Acid via Sulfonating Agent Sulfonation

**Figure 4:**
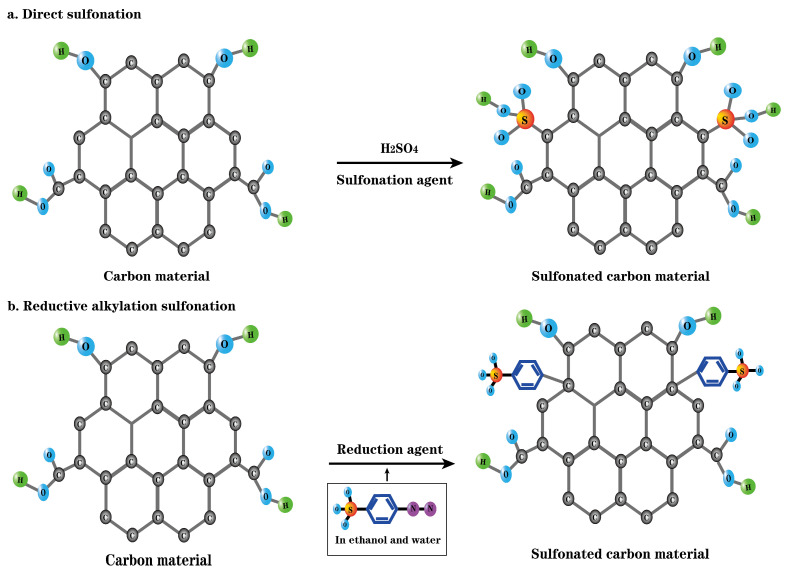
Sulfonation mode of carbon-based solid acid: (**a**) direct sulfonation; (**b**) reductive alkylation sulfonation [103].

Sulfonating agent sulfonation is the most commonly used method for the acidification of carbon-based solid acid catalysts [78,79,80]. Its works by combining sulfonic acid groups with polycyclic aromatic carbon rings in the form of covalent bonds through the reaction of sulfonating agents with carbon-based materials. The carbon-based solid acid produced via this acidification method is tightly anchored by the acidic group and is not easily lost during the catalytic reaction. At present, there are two kinds of carbon-based solid acid processes in sulfonation acidification: direct sulfonation [101] and reductive alkylation [102]. The mechanisms of the two sulfonation methods are shown in Figure 4 [103]. The introduction of sulfonic acid groups to carbon materials using both methods is mainly achieved through grafting (covalent connection, electrochemical, chemical reduction, and reductive alkylation) or co-condensation and subsequence oxidation method [10,104]. The common sulfonating agents mainly include concentrated sulfuric acid [81,82], arylsulfonic acid [87,88], and halogenated sulfonic acid [83,84]. 

Concentrated sulfuric acid is often used in the chemical industry because of its high acidity and stable performance. For this reason, many researchers use concentrated sulfuric acid as a sulfonating agent to prepare carbon-based solid acids in order to obtain a solid acid with the performance of concentrated sulfuric acid. Araujo et al. [105] used acai stone as the carbon precursor and concentrated sulfuric acid as the sulfonating agent. Through detection, characterization, and evaluation of transesterification performance, carbonization was carried out at 400 °C for 1 h, and then carbonization was performed in concentrated sulfuric acid for 25 °C acidification for 2 h, producing the best solid acid material. It is worth mentioning, from this study, that the content of sulfonic acid groups in the carbon-based solid acid produced at a sulfonation temperature of 100 °C was three times that produced at a sulfonation temperature of 25 °C, but the catalytic performance of the two was similar in the transesterification reaction. In addition, although solid acid has considerable catalytic performance in the initial stage, its stability is poor, and the catalytic performance is reduced by nearly half after a second use. This may be related to the loss of the sulfonic acid group due to insufficient binding.

Although concentrated sulfuric acid performs well, its corrosiveness causes equipment loss and corresponding safety problems. Therefore, some researchers have proposed replacing concentrated sulfuric acid with less corrosive sulfonating agents [85,86,89] such as arylsulfonic acid and halogenated sulfonic acid to prepare carbon-based solid acids. Researchers [106] used defatted oil cake and methanesulfonic acid as raw materials to prepare sulfonated carbon-based solid acids, which performed well in catalyzing cellulose hydrolysis. After a series of analyses, the results showed that the synthesis temperature and acid concentration had a significant effect on the surface properties of carbon-based solid acids, while the synthesis time had no significant effect on the carbon-based solid acids. Farabi et al. [107] used palm kernel shells and bamboo as carbon precursors and chlorosulfonic acid as a sulfonating agent to prepare a carbon-based solid acid by first carbonizing and then sulfonating. The resulting solid acid greatly increased the conversion rate of palm fatty acid distillate (PFAD) into biodiesel. 

To summarize, the acidity and function of carbon-based solid acids obtained via different sulfonation methods are slightly different. The acidity of a carbon-based solid acid after direct sulfonation is usually weaker than that of the sulfonating agent itself. Because these solid acids are relatively stable, they can be used multiple times without any additional treatment. The acidity of the solid acids obtained via indirect sulfonation is generally stronger than that of the sulfonation agent. In addition, compared with the direct sulfonation method, which involves the use of corrosive hazardous chemicals as the sulfonation agent, the sulfonation agent used in the indirect method is generally safer. However, this kind of catalyst easily loses the sulfonate group and deactivates in catalytic reactions.

### 3.2. Preparation of Carbon-Based Solid Acid via Acidification of Phosphoric Acid

**Figure 5:**
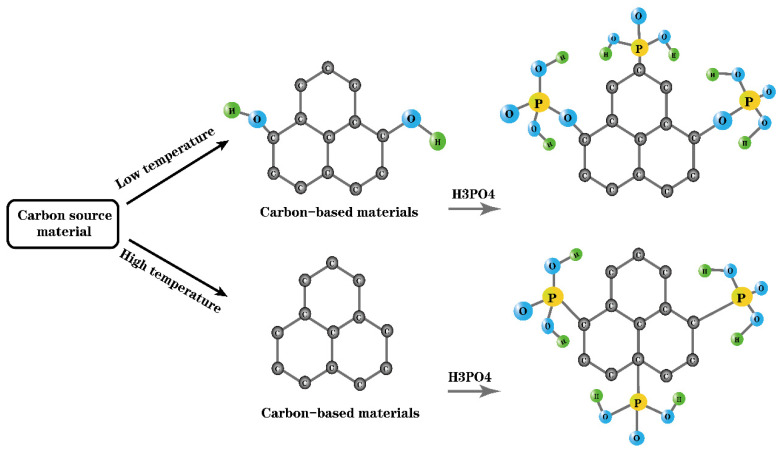
Phosphorylation mechanism of ordered mesoporous carbon at different carbonization temperatures [109].

While studying the use of arylsulfonic acid and halogenated sulfonic acid instead of concentrated sulfuric acid to prepare carbon-based solid acids, some researchers have proposed the use of the less-corrosive and nonpolluting phosphoric acid as an acidifying agent [90,91,92]. It has been found that in the process of preparing carbon-based solid acid via the acidification of phosphoric acid, the carbonization temperature has an impact on the combination of phosphoric acid and carbon-based materials [93,94]. When the carbonization temperature is low, there are many hydroxyl groups on the surface of a carbon-based material. Phosphoric acid can be directly bonded to a carbon-based material through C-PO_3_, or it can be attached to the surface of a carbon-based material in the form of C-O-P through the dehydration of the hydroxyl group. At a relatively high carbonization temperature, the surface of a carbon-based material lacks active hydroxyl groups, and phosphoric acid is mainly bound in the form of C-PO_3_, so has higher stability [108]. This specific situation is shown in Figure 5 [109]. For this reason, different carbonization temperatures lead to large differences in the properties of the final solid acid.

Wang et al. [109] used phosphoric acid to acidify ordered mesoporous carbon materials prepared at different carbonization temperatures, and then they catalyzed the esterification reaction of oleic acid and methanol. After comparison, it was found that the carbon-based solid acid prepared at a higher carbonization temperature had catalytic activity and stability that were superior to those of carbon-based solid acids prepared at lower carbonization temperatures. Fulvio et al. [110] used a triblock polymer as a template and a mixture of resorcinol, formaldehyde, and phosphoric acid as a carbon precursor to prepare phosphorylated mesoporous carbon via a soft template method. In catalyzing the dehydration reaction of isopropanol, the carbon-based solid acid could significantly reduce the conversion temperature. 

In recent years, researchers have found that the oxygen-containing components in phosphoric acid have a good affinity with uranyl ions, and phosphoric acid has become a high-quality adsorbent for uranium-containing wastewater. Hu et al. [111] used bamboo powder as a carbon precursor, mixed it with phosphoric acid, and carbonized it in a tube furnace to prepare a phosphorylated carbon material. The material had an adsorption capacity of 229.2 mg·g^−1^ in the test of nuclear wastewater adsorption of uranium. Sun et al. [112] used chitosan as a carbon precursor, which was carbonized via the hydrothermal method, and then acidified with phosphoric acid to obtain the desired acidic material. The material exhibited good adsorption selectivity and strong adsorption capacity for uranium in aqueous solution. On this basis, researchers [113] made a honeycomb porous acidic carbon material using orange peel as the carbon precursor, carbonizing it in a tube furnace, and then acidifying it with phosphoric acid. In their experiment on treating nuclear wastewater, the material showed an adsorption capacity of 552.6 mg·g^−1^ of uranium, and after five reuses, its adsorption capacity was still 90.5% the of the original. Cai et al. [114] placed a small amount of graphene–chitosan composite in triethyl phosphite, and heated it in an oil bath at 150 °C for 36 h to obtain the desired phosphorylated carbon-based material, which could adsorb uranium up to 779.44 mg·g^−1^. Based on the above studies, it can be seen that in experiments of phosphorylated carbon-based solid acid in the treatment of nuclear wastewater, the carbon precursor material has a significant impact on the adsorption capacity of uranium.

### 3.3. Preparation of Carbon-Based Solid Acid via Acidification of Heteropoly Acid

Carbon materials have a large specific surface area (>100 m^2^·g^−1^) and rich pore structures (micropores, mesopores, and macropores); hence, some researchers think that they can be used as carriers to load acidic substances to prepare solid acids. At present, many studies have been conducted on solid acids loaded with heteropoly acids, and their preparation methods mainly include the impregnation method [95,96] and the adsorption method [23,115]. The impregnation method involves placing a carbon-based material in a certain concentration of heteropoly acid solution, stirring it at a constant temperature for several hours, evaporating the excess water in a water bath, and then drying it in an oven. The adsorption method involves placing a carbon-based material in a heteropoly acid solution of a certain concentration, filtering out the mother liquor after stirring and refluxing, and drying the wet sample in an oven. The acid adsorption amount can be calculated by the change in acid substance content in the acidic solution before and after adsorption [97]. In 2019, Ghubayra et al. [116] studied the effect of three Keggin-type heteropoly acids loaded on activated carbon on the oxidative desulfurization of diesel.

A small amount of activated carbon was placed in heteropoly acid aqueous solution via the impregnation method; the mixture was magnetically stirred at 40 °C for 3 h and then dried in an oven at 100 °C overnight to obtain a black powder catalyst. The catalytic activities of the three Keggin-types heteropoly acids in the desulfurization reaction of diesel oil were H_3_PMo_12_O_40_ > H_3_PW_12_O_40_ > H_4_SiW_12_O_40_ in descending order, among which H_3_PMo_12_O_40_ with the highest catalytic activity could completely remove benzothiophene in diesel at 60 °C. The catalytic activity of the catalyst remained unchanged after repeated recycling. Based on the above research, a team [117] further explored the relationship between the catalytic activity of catalyst and properties of the carrier. Using activated carbon with different acid–base properties as the carriers, a heteropoly acid was attached to the activated carbon in the same way. Through the analysis of catalytic activity and characterization, it was found that the Keggin structure of the heteropoly acid decomposed when the pH value of the activated carbon was in the range of 5.5–10.1, and the decomposed catalyst had better catalytic performance than the original catalyst. Chen et al. [118] used ordered mesoporous carbon prepared via the template method as a carrier to prepare carbon-based solid acid via loading dodecyl tungstophosphoric acid (H_3_PW_12_O_40_). The solid acid exhibited good catalytic performance in the microwave esterification of acetic acid and isoamyl alcohol. Prado et al. [119] used wood slag as a raw material for the carbon carrier and loaded dodecaphosphomolybdic acid (H_3_PMo_12_O_40_) via the impregnation method to prepare a carbon-based solid acid. When the solid acid catalyzed the esterification reaction of lauric acid and methanol, the yield of methyl laurate significantly increased. In the catalyst reuse and leaching tests, when the catalyst was directly reused without pretreatment, the activity of the catalyst dropped significantly. 

Based on the comprehensive studies conducted on the preparation of solid acids via the acidification of heteropoly acids, it has been found that acid strength and amount are important factors affecting the catalytic activity of solid acids. In general, when heteropoly acids are loaded on carbon-based materials, the acid strength and total acid content decrease [98,99,100]. The main factors affecting the acid strength and amount are [13]: (1) the nature of the carrier, which may change the structure of the heteropoly acid and thus affect the acid strength and acid amount; (2) the acid load, acid strength, and total acid amount. The acid amount increases with the increase in the load, which is due to the fact that the acidity of the heteropoly acids adsorbed on the surface of activated carbon is not uniform, the heteropoly acids that enter the strong adsorption point first are adsorbed more firmly, and the acidity is weaker. However, the adsorption firmness of a heteropoly acid absorbed by multilayer activated carbon is weakened and the acidity is stronger. (3) The types of heteropoly acids, different types of heteropoly acids, their acid strength, and the way they combine with carbon-based materials are also quite different. In addition, during the preparation process, the concentration of the impregnating solution, the specific surface area and pore size of the carbon-based material, the drying temperature, and time all affect the activity of a catalyst [120].

### 3.4. Preparation of Carbon-Based Solid Acid by Nitric Acid Acidification

In addition to sulfonating agent sulfonation, phosphoric acid acidification, and heteropoly acid acidification, there is also a method for preparing carbon-based solid acids called nitric acid acidification, which is not commonly used due to its difficult preparation process. Nitric acid acidification mainly uses nitric acid as an oxidant to oxidize carbon-based materials to introduce (-carbonyl) acid groups to achieve the purpose of acidification. Traditional nitric acid oxidation has two major disadvantages: (1) Under normal pressure, the boiling point of concentrated nitric acid is about 122 °C. In the liquid-phase oxidation of carbon-based materials, the limiting oxidation temperature is the same as the boiling point temperature, so the oxidation effect of carbon materials cannot be improved by increasing the temperature [121]. (2) In the process of stirring, the carbon fragments stripped via oxidation are continuously detached from the carbon-based material and then adsorbed, so that a large number of carbon fragments attach to the oxidized carbon-based material. [122]. In addition, harmful gases such as NOx are produced during the oxidation of carbon-based materials with nitric acid, and the waste acid after the reaction is difficult to recycle. In order to improve the shortcomings of traditional nitric acid oxidation, Xia et al. [123] invented a gas-phase oxidation method, which has two advantages. First, gas-phase oxidation is not affected by the boiling point of nitric acid, the oxidation effect can be improved by increasing the temperature, which reduces the consumption of nitric acid. Second, the carbon fragments do not easily fall off during gas-phase oxidation. When the degree of oxidation increases, the number of carboxyl active sites increases, thereby improving the overall catalytic activity. In addition, Durán et al. [124] treated activated carbon with nitric acid and a sulfonating agent to prepare two carbon-based solid acids. In the ring-opening reaction of ethanol to epoxide oxidation of styrene, it was found that the catalytic performance of the sulfonating agent was higher than that of nitric acid.

## 4. Summary and Outlook

With the emphasis on achieving a low-carbon society, environmentally friendly development, and *green chemistry*, carbon-based solid acids have gradually become the key acid catalyst research objects of owing to their ease of preparation, strong acidity, and high catalytic activity. Different methods of carbonizing carbon-based solid acids lead to changes in their structures and functions; thus, researchers must first consider the use of carbon-based solid acid while preparing carbon-based solid acids and then choose the most appropriate carbonization process according to specific uses. In addition, the acidification process is a critical step related to the various features in carbon-based solid acid. The selection of the acid reagent for acidification determines the acidity strength and acid catalytic site characteristics of the resulting solid acid. This paper provided a systematic overview of the research progress on carbon-based solid acid catalysts and their preparation methods, including one-step carbonization, two-step carbonization, precise targeting method carbonization, followed by sulfonating agent acidification, phosphoric acid acidification, heteropoly acid acidification, and nitric acid acidification. Using the different preparation methods, the produced carbon-based solid acids have good chemical catalytic activity. However, the progress in the preparation of carbon-based solid acid catalysts is still in an exploratory stage. And, the remaining problems can be summarized in four points: 

(1) The stability and uniformity of carbon-based solid acids cannot be guaranteed. This problem is relevant to biomass, which is often used as the carbon precursor material of carbon-based solid acids. Biomass is mainly composed of cellulose, hemicellulose, and lignin. The contents of these components affect the temperature and method of carbonization, thus changing the functional properties of carbon-based solid acids. This problem is key to the commercial development of carbon-based solid acids. Therefore, it is necessary to deeply study the functions and properties of carbon-based solid acids prepared through different carbonization methods and acidification types, summarize the rules and changes, and then establish a corresponding theoretical basis to lay the foundation for further exploration of the stable preparation of carbon-based solid acids from biomass.

(2) As far as the current research on carbon-based solid acids is concerned, most of them are biased toward the acidity of the solid acid and the stability of its catalytic performance. Less research has focused on the anchoring method between the acidic groups on the acid sites of carbon-based solid acids and carbon materials. Therefore, it is necessary to continue exploring the influence of different carbonization methods and acidification types on the basic structure of carbon-based solid acids and to guide the development of the optimal preparation process through revealing the degree of anchoring and modification of the acidic groups in carbon materials using different treatment methods, to achieve stable, selective, and scalable commercial catalyst production standards. 

(3) Carbon materials obtained via direct pyrolysis often have a small specific surface area and pore volume, which limit its application in many fields. At present, some studies have applied activation methods on the basis of direct pyrolysis to further optimize the pore structure properties and improve application performance. Commonly used activation media are KOH, K_2_CO_3_, NaOH, and ZnCl. However, these researchers have seldom studied the activation mechanism, and some activation media may corrode the equipment and increase the process flow. Therefore, it is necessary to explore the activation mechanism of different activation media to guide the selection of activation media when preparing carbon-based solid acids.

(4) Most of the preparation methods used in the current research require high-temperature carbonization and high-concentration acid solution impregnation, which cause the generation of by-products such as harmful gases and waste acid solutions. The evaluation of these aspects continues to be overlooked in the development of novel carbon-based solid acids. Therefore, further research is suggested to improve the environmental aspect of the technology in order to remove harmful by-products produced in the preparation process to ensure high-quality catalytic performance.

Carbon-based solid acids are a part of material science. Small changes in their atomic structure may lead to profound changes in the properties of solid acids, and the relationships between structure and properties are quite complex [125]. In the field of material science, machine learning is a set of methods used for learning the correlation between patterns (atomic structure) and numbers (material properties, such as acid strength, conductivity, etc.) [126,127,128]. At present, machine learning has achieved notable results in the synthesis of new materials, the analysis of material characterization data, and the prediction of catalytic reactions [129,130,131]. Therefore, if a data model can be established through machine learning to reveal the influence of carbon-based solid acid catalyst materials on the structure changes of acid sites, acid groups, raw materials, and carbon-based solid acids under the influence of different preparation conditions, so as to clearly analyze their preparation and synthesis mechanism. Finally, a stable, cheap, and highly selective carbon-based solid acid catalytic material will be developed, and the preparation ideas and application prospects of carbon-based solid acid will be expanded.

## Figures and Tables

**Table 1:** Properties and functions of carbon-based solid acids produced with different carbonization methods.

Raw Material	Preparation Method	Characterization of Solid Acids			Function	Yield	Conversion Rate	Ref
		Acid	S_BET_ m^2^/g	V_P_ m^3^/g		%	%	
		Group						
		mmol/g						
**One-Step Direct Carbonization**								
Rice husk and sulfuric acid	At 240 °C for 2 h	1.45	-	-	Synthetic biodiesel	-	95.4	[25]
Vinasse and sulfuric acid	At 20 °C for 1.4 h	0.89	26.25	-	Acetate esterification	-	97.6	[26]
Durian peel and sulfuric acid	At 100 °C for 2 h	1.22	-	-	Synthetic biodiesel	-	81.67	[27]
Microcrystalline cellulose and sulfuric acid	At 125 °C for 1 h	1.31	-	-	Oleic acid esterification	-	80	[29]
**Step carbonization**								
Sucralose and sulfuric acid	Carbonization at 300 °C for 0.5 h, sulfonation at 150 °C for 15 h	0.67	0.31	-	Preparation of lignin and glucose	33.9 6.9	-	[73]
Coconut shell and sulfuric acid	Carbonization at 450 °C for 1 h, sulfonation at 130 °C for 16 h	1.27	10.16	-	Preparation of glucose	91	-	[74]
Waste coffee powder and sulfuric acid	Carbonization at 500 °C for 5 h, sulfonation at 60 °C for 3 h	-	15.39	-	Fenton oxidative degradation of methyl orange	-	70.2	[75]
Paper scraps and sulfuric acid	Carbonization at 400 °C for 6 h, sulfonation at 150 °C for 15 h	0.67	402.1	-	Synthesis of n-butyl levulinate	90.6	-	[76]
Corncob residue and sulfuric acid	Carbonization at 400 °C for 3 h, sulfonation at 150 °C for 10 h	0.68	282.94	-	Preparation of sugar aldehyde	73.64	-	[77]
Potato skin and sulfuric acid	Carbonization at 450 °C for 1 h, sulfonation at 180 °C for 8 h	1.6	827.7	0.92	Synthetic biodiesel		97.2	[40]
Bamboo powder and sulfuric acid	Carbonization at 400 °C for 1 h, sulfonation at 25 °C for 1 h	1.80	1.17	0.00061	Synthetic biodiesel		97.31	[44]
Pampas grass and sulfuric acid	Carbonization at 400 °C for 6 h, sulfonation at 150 °C for 12 h	2.3	278	-	Synthetic biodiesel	98.9	99.1	[46]
**Carbonization via precise target method**								
Lignin and sulfuric acid	Hydrothermal carbonization at 240 °C for 10 h and sulfonation at 180 °C for 12 h.	1.2	-	-	Preparation of glucose	59.1	-	[53]
Diosgenin by-products and chlorosulfonic acid	Hydrothermal carbonization at 200 °C for 10 h and sulfonation for 4 h.	1.41	5.17	0.0125	Preparation of diosgenin	22.4	-	[54]
Orange peel and sulfuric acid	Hydrothermal carbonization at 180 °C for 12 h and sulfonation at 80 °C for 2 h.	1.85	4.78	0.026	Esterification of oleic acid and citric acid	-	92.8 81.3	[58]
Glucose and sulfuric acid	200 °C hydrothermal carbonization for 6 h, 180 °C sulfonation for 5 h	2.6	-	-	Preparation of ethyl levulinate	67.1	-	[60]
Sucrose and sulfanilic acid	Template carbonization at 160 °C for 6 h and sulfonation at room temperature for 20 h.	2.18	547	0.47	Production of ethyl acetate	-	95	[50]
Formaldehyde, resorcinol and sulfuric acid	Carbon precursor prepared with soft template method is carbonized at 800 °C for 2 h and sulfonated at 140 °C for 20 h	0.44	444	1.46	Glycerol esterification	-	95	[62]
Lignin and acrylic acid	Template carbonization at 900 °C for 2 h and sulfonation at 190 °C for 16 h	-	1197.1	0.63	Preparation of 5-hydroxymethylfurfural	96	-	[61]

**Table 2:** Properties and functions of carbon-based solid acids with different acidification types.

Raw Material	Acidizing Method	Acidity of Solid Acids			Function	Yield	Conversion Rate	Ref
		SO_3_H Group mmol/g	PO_3_H_2_ Group mmol/g	Heteropoly Acid mmol/g		%	%	
**Sulfonic Acid Acidification**								
Chicken bone and p-aminobenzenesulfonic acid	P-aminobenzenesulfonic acid sulfonated at 80 °C for 12 h	2.33			Synthesis of 5-ethoxymethylfurfural	68.6	-	[78]
Lignin, PVC and p-aminobenzenesulfonic acid	P-aminobenzenesulfonic acid sulfonated at 80 °C for 0.5 h	0.86			Synthetic sugar alcohol	84.3	-	[79]
Orange peel pectin and sulfuric acid	Sulfuric acid sulfonation at 170 °C for 6 h	1.35	-	-	Synthetic sugar aldehyde	80.4	100	[80]
Activated carbon and sulfuric acid	Sulfuric acid sulfonation at 150 °C for 4 h	0.64			Synthetic biodiesel	94.2	95.2	[81]
Cellulose, lignin and sulfuric acid	Sulfuric acid sulfonation at 150 °C for 6 h	0.74			Preparation of reducing sugar	60		[82]
Sunflower seed hull and chlorosulfonic acid	Sulfonation in chlorosulfonic acid at 25 °C for 2 h	-	-	-	Itaconic acid esterification	77	100	[83]
Cotton ginning waste and chlorosulfonic acid	Sulfonation of chlorosulfonic acid for 9 h at room temperature	1.89			Preparation of 5-hydroxymethylfurf-ural	71.7	76.74	[84]
β-Cyclodextrin and 2-hydroxyethanesulp-honic acid	2-hydroxyethanesulp-honic acidsulfonated at 180 °C for 4 h	-			Preparation of succinic acid	81.2		[85]
Glucose and 2-hydroxyethanesulp-honic acid	2-hydroxyethanesulp-honic acidsulfonated at 180 °C for 4 h	2.1			Preparation of reducing sugar	99.8		[86]
Glucose and p-toluenesulfonic acid	p-toluenesulfonic acid sulfonated at 180 °C for 24 h	0.68			Hydrolyzed microcrystalline cellulose		30.9	[87]
Sucralose and p-toluenesulfonic acid	p-toluenesulfonic acid sulfonated at 180 °C for 24 h	-			Preparation of reducing sugar	67.6		[88]
Undaria pinnatifida and p-toluenesulfonic acid	p-toluenesulfonic acid sulfonated at 200 °C for 3 h	0.234	-	-	Acetate esterification	81.9	98.2	[89]
**Acidification of phosphoric acid**								
Antibiotic residues and phosphoric acid	Ultrasonic immersion with phosphoric acid for 6 h		3.05		Pyrolysis of catalytic waste mixed cloth	-	-	[90]
Municipal sludge and phosphoric acid	Ultrasonic immersion with phosphoric acid for 3 h		0.82		Production of levoglucone	19.6		[91]
Bagasse and phosphoric acid	Soak in phosphoric acid for 2 h	-	-	-	Production of levoglucone	18.1	-	[92]
Birch and phosphoric acid	Soak in phosphoric acid for 6 h	-	-	-	Production of levoglucone	20	-	[93]
Activated carbon and phosphoric acid	Phosphoric acid impregnation	-	0.385	-	Synthetic dimethyl ether	-	47	[94]
**Heteropoly acid acidification**								
Activated carbon and phosphotungstic acid	Phosphotungstic acid impregnation	-	-	31	Synthetic tert-myl thyl ether	-	18.8	[95]
Activated carbon and silicotungstic acid	Silicotungstic acid immersion 72 h	-	-	20	Preparation of glucose	94	-	[96]
Carbon nanotubes and Phosphomolybdic Acid	Phosphomolybdic acid impregnation	-	-	15	Synthetic biodiesel	86.7	98.2	[97]
Activated carbon and12- tungstophosphoric acid	12-tungstenphosphoric acid immersion for 12 h	-	-	50	Synthesis of 5-hydroxymethylfurf-ural	99.3	100	[98]
Bagasse and phosphotungstic acid	Phosphotungstic acid impregnation	-	-	20	Preparation of fructose	56.2	-	[99]
Activated carbon and H_6_PV_3_MoW_8_O_40_	H_6_PV_3_MoW_8_O_40_ impregnation	-	-	35	Synthetic biodiesel	-	91.3	[100]
